# Ultra-restrictive red blood cell transfusion strategies in extensively burned patients

**DOI:** 10.1038/s41598-024-52305-y

**Published:** 2024-02-03

**Authors:** Yiran Wang, Zhikang Zhu, Deqing Duan, Wanting Xu, Zexin Chen, Tao Shen, Xingang Wang, Qinglian Xu, Hongyan Zhang, Chunmao Han

**Affiliations:** 1 Department of Burns & Wound Care Center, The Second Affiliated Hospital of Zhejiang University College of Medicine, Hangzhou, 310009 China; 2 The Key Laboratory of the Diagnosis and Treatment of Severe Trauma and Burn of Zhejiang Province, Hangzhou, China; 3 Department of Burns, The First Affiliated Hospital of Nanchang University, Nanchang, China; 4 Department of Burn Injury, The First Affiliated Hospital of Anhui Medical University, Hefei, China; 5 Center of Clinical Epidemiology & Biostatistics, Department of Scientific Research, The Second Affiliated Hospital of Zhejiang University College of Medicine, Hangzhou, China

**Keywords:** Haematological diseases, Trauma

## Abstract

In recent years, due to the shortage of blood products, some extensive burn patients were forced to adopt an “ultra-restrictive” transfusion strategy, in which the hemoglobin levels of RBC transfusion thresholds were < 7 g/dl or even < 6 g/dl. This study investigated the prognostic impacts of ultra-restrictive RBC transfusion in extensive burn patients. This retrospective multicenter cohort study recruited extensive burns (total body surface area ≥ 50%) from three hospitals in Eastern China between 1 January 2016 and 30 June 2022. Patients were divided into an ultra-restrictive transfusion group and a restrictive transfusion group depending on whether they received timely RBC transfusion at a hemoglobin level < 7 g/dl. 1:1 ratio propensity score matching (PSM) was performed to balance selection bias. Modified Poisson regression and linear regression were conducted for sensitive analysis. Subsequently, according to whether they received timely RBC transfusion at a hemoglobin level < 6 g/dl, patients in the ultra-restrictive transfusion group were divided into < 6 g/dl group and 6–7 g/dl group to further compare the prognostic outcomes. 271 eligible patients with extensive burns were included, of whom 107 patients were in the ultra-restrictive transfusion group and 164 patients were in the restrictive transfusion group. The ultra-restrictive transfusion group had a significantly lower RBC transfusion volume than the restrictive transfusion group (11.5 [5.5, 21.5] vs 17.3 [9.0, 32.5] units, *p* = 0.004). There were no significant differences between the two groups in terms of in-hospital mortality, risk of infection, hospital length of stay, and wound healing time after PSM or multivariate adjustment (*p* > 0.05). Among the ultra-restrictive transfusion group, patients with RBC transfusion threshold < 6 g/dl had a significantly higher hospital mortality than 6–7 g/dl (53.1% vs 21.3%, *p* = 0.001). For extensive burn patients, no significant adverse effects of ultra-restrictive RBC transfusion were found in this study. When the blood supply is tight, it is acceptable to adopt an RBC transfusion threshold of < 7 g/dL but not < 6 g/dL.

## Introduction

Extensive burns result in a long disease course, high infection risk, large number of procedures, and high risk of mortality. Because of factors such as blood loss from the trauma itself, multiple operations, gastrointestinal bleeding, and the decreased erythrocyte production associated with nutritional deficiencies and systemic inflammation, patients with extensive burns are susceptible to severe and prolonged anemia^1–3^. Red blood cell (RBC) transfusion is an indispensable treatment to correct anemia in extensive burn patients which can increase hemoglobin level and blood volume in the short term, thus improve oxygen extraction and stabilize the internal environment^4,5^. However, due to the possible adverse effects of transfusion including infection, pulmonary edema, immune suppression, iron overload, and microcirculatory alterations, as well as the limited blood resources, the RBC transfusion strategies for extensive burns must be selected with caution^6–8^.

To date, the clinical studies about RBC transfusion strategies for various populations are mainly focused on the comparison of prognostic outcomes between restrictive transfusion strategy (hemoglobin threshold 7.0 g/dl to 8.0 g/dl) and liberal transfusion strategy (hemoglobin threshold 9.0 g/dl to 10.0 g/dl). Most conclusions showed no difference between the two strategies in the risk of mortality and complications^6,9^. For burn patients, a multicenter randomized prospective trial (RCT) named Transfusion Requirement in Burn Care Evaluation (TRIBE) showed that the two transfusion strategies have no significant effect on mortality, wound healing time (WHT), bloodstream infection (BSI) rate, and other prognostic outcomes in patients with TBSA burned ≥ 20%^7^. Alternatively, other studies suggested the restrictive transfusion strategy significantly reduced the incidence of infection and mortality^10–12^. Based on the above research findings, and considering the saving of blood resources, the restrictive transfusion strategy was recommended by current clinical guidelines for burn patients^7,13,14^.

However, though adopting the restrictive transfusion strategy, the treatment of extensive burn patients still required large amounts of RBC products. In the period of blood resources absence, RBC products were still unable to meet the needs of all patients^15–17^. Some extensive burn patients were forced to adopt an “ultra-restrictive” transfusion strategy, in which RBC transfusion thresholds were < 7 g/dl or even < 6 g/dl. The COVID-19 pandemic was likely to increase the number of patients undergoing ultra-restrictive transfusions in recent years. This study analyzed the prognostic impact of the ultra-restrictive transfusion strategy in extensive burn patients to probe the feasibility of further reducing RBC transfusion thresholds in extensive burns and promote the rational allocation of limited blood resources.

## Study design and methods

### Study participants

This retrospective study included patients at three hospitals. All three hospitals are tertiary care centers with specialized burn units and provide standardized treatment of extensive burn patients, including rapid airway establishment, early fluid resuscitation, scab excision, wound coverage, maintenance of organ function, infection control, and nutritional support therapy. Fluid resuscitation treatments were guided by the Third Military Medical University Fluid Resuscitation Formula, and adjusted according to individual conditions^18^. RBC transfusions were routinely used as treatment for anemia in which the transfusion thresholds were adjusted based on the current RBC reserves. Under conditions of adequate RBC reserves, a restrictive transfusion strategy was commonly employed, setting the transfusion threshold at 7–8 g/dl. In times of blood resource scarcity, RBC resources were coordinated by the hospitals' transfusion department, with the RBC transfusion threshold being lowered according to the actual degree of resource shortage. Intraoperative RBC transfusions were adjusted according to the intraoperative bleeding and hemodynamics of patients and were not limited by transfusion thresholds.

**Figure 1:**
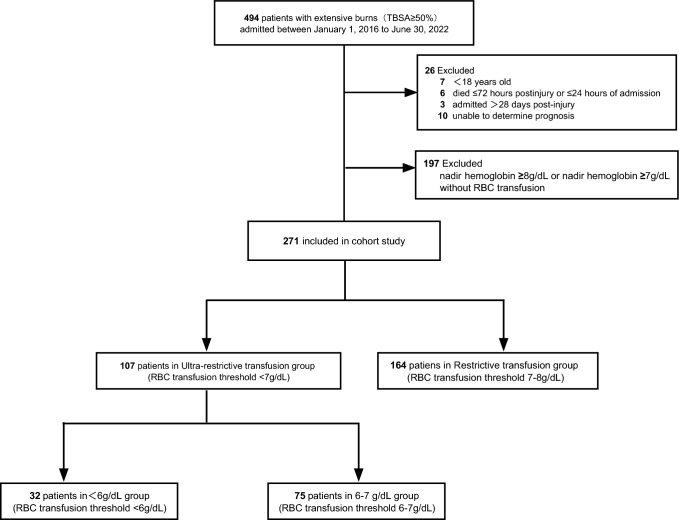
Flowchart of participants selected and data analysis. *TBSA* total body surface area, *RBC* red blood cell.

The inclusion criteria were as follows: (1) patients accepted inpatient treatment from 1 January 2016 to 30 June 2022 in the burn units of the three hospitals; (2) patients with TBSA burned ≥ 50%; (3) patients with complete electronic medical records. The exclusion criteria were as follows: (1) patients < 18 years of age; (2) pregnant patients; (3) patients who died within 72 h post-injury or within 24 h of admission; (4) patients admitted > 28 days post-injury; (5) patients discharged for personal reasons and patients whose prognoses could not be determined; (6) patients with recorded pre-existing anemia; (7) patients with a nadir hemoglobin level ≥ 8 g/dl; (8) patients with a nadir hemoglobin level ≥ 7 g/dl and without RBC transfusion during hospitalization (Fig. 1).

We followed the Strengthening the Reporting of Observational Studies in Epidemiology guidelines for conducting this retrospective cohort study. The research was approved by the Institutional Ethical Committee of the Second Affiliated Hospital of Zhejiang University College of Medicine (I20221060), the Institutional Ethical Committee of the First Affiliated Hospital of Nanchang University ((2023)CDYFYYLK(01-043)), and the Institutional Ethical Committee of the First Affiliated Hospital of Anhui Medical University (PJ 2023-10-12). All of the Institutional Ethical Committees waived the requirement for informed consent due to the observational, retrospective nature of this study.

### Data collection

All information was collected using the same checklist from the electronic medical record systems of different hospitals. The following demographics and clinical characteristics were collected: gender, age, BMI (body mass index), TBSA burned, full thickness burned area, type of burn injury, inhalation injury, pulmonary edema, admission Multiple Organ Dysfunction Score (MODS), admission Acute physiology and chronic health evaluation II (APACHE II) score, revised Baux (rBaux) score, underlying disease, admission to the first operation, operation times and mechanical ventilation. Pulmonary edema was defined as the radiologically diagnosed accumulation of pulmonary interstitial fluid within 7 days after admission. The admission MODS and admission APACHE II scores were calculated based on parameters recorded during the first and second 24-h periods after admission^19,20^. rBaux scores were calculated as age + TBSA burned% + 17 (if inhalation injury)^21^. The hemoglobin values along with the time of each measurement, as well as the timing, volume, and adverse effects of each RBC transfusion during hospitalization were also collected. Nadir hemoglobin was defined as the lowest hemoglobin value measured during hospitalization. Preoperative hemoglobin was defined as the most recent hemoglobin value measured within 2 days before surgery, and postoperative hemoglobin was defined as the most recent hemoglobin value measured within 2 days after surgery. The hemoglobin difference between pre-and postoperative was defined as the value of preoperative hemoglobin minus postoperative hemoglobin. Hemoglobin before RBC transfusion was defined as the most recent hemoglobin value measured before each RBC transfusion event.

The prognostic variables were collected and included: in-hospital mortality, hospital length of stay (LOS), WHT, BSI, sepsis, wound infection, and catheter-related infection. WHT was defined as the time point at which wound closure by re-epithelisation reached 90%, or 7 days after the last excision and grafting treatment^7^. BSI, wound infection, and catheter-related infection were defined based on the isolation of bacteria or fungi from at least one culture^22^. Sepsis was defined as suspected infection and Sequential [Sepsis-related] Organ Failure Assessment(SOFA) score ≥ 2^23^.

### Study design

‘Ultra-restrictive transfusion’ was defined as the absence of RBC transfusion for > 2 days in a patient with a hemoglobin level < 7 g/dl—below the level used in the restrictive transfusion strategy^7,13,14^. Patients who had experienced one or more ‘ultra-restrictive transfusion’ were assigned to the ultra-restrictive transfusion group, while other patients were assigned to the restrictive transfusion group. Subsequently, patients in ultra-restrictive transfusion group were stratified into < 6 g/dl group and 6–7 g/dl group based on whether they experienced the absence of RBC transfusion for > 2 days with a hemoglobin level < 6 g/dl.

The primary outcome measure of prognostic impact was in-hospital mortality. Secondary outcome measures included the incidences of BSI, sepsis, wound infection, and catheter-related infection, as well as hospital LOS and WHT determined in surviving patients (Fig. 1).

### Statistical analysis

Multiple imputation was used to impute missing values, assuming randomly missing values, and using 10 imputed data sets. According to the Kolmogorov–Smirnov test, normally distributed continuous variables were expressed as medians ± standard deviation (SD); differences between groups were identified using the t-test. Non-normally distributed continuous variables were expressed as medians with interquartile ranges; differences between groups were identified using the Mann–Whitney *U* test. Categorical variables were expressed as numbers (percentages); comparisons were performed using the chi-squared test or Fisher’s exact test, as appropriate. Violin plots were used to present the full distribution of the RBC transfusion volumes. To minimize the impacts of potential confounders and selection bias, propensity score matching (PSM) was used to compensate for differences in baseline patient characteristics between two group. A propensity score was calculated using logistic regression, and 1:1 patient matching was performed using the nearest-neighbor matching method without replacement. 13 baseline characteristics, respectively gender, age, BMI, TBSA burned, type of burn injury, inhalation injury, pulmonary edema, admission MODS score, underlying disease, operation times, mechanical ventilation use, admission hemoglobin and hospital were matched and caliper radius equal to a standard deviation of 0.1 was set. Standardized differences were estimated before and after matching to evaluate balance, and a value of less than 0.2 indicated a balance between groups^24^. Kaplan–Meier and a log-rank test were used to compare survival between the two groups. Modified Poisson regression and linear regression were also conducted for sensitive analysis. Baseline characteristics whose *p*-values between groups < 0.1 were included in the regressions for adjustment using the Enter method. Two-tailed *p* values < 0.05 were considered indicative of statistical significance. All statistical analyses were performed using SPSS software (version 27) and R software (4.2.2).

## Results

### Demographics and clinical characteristics

Among the 494 patients admitted to the three hospitals between 1 January 2016 and 30 June 2022, 223 were excluded: 7 patients were < 18 years of age, 6 patients died within 72 h post-injury or within 24 h of admission, 3 patients were admitted > 28 days post-injury, 10 patients unable to determine prognosis, and 197 patients with a nadir hemoglobin level ≥ 8 g/dl or a nadir hemoglobin level ≥ 7 g/dl without RBC transfusion during hospitalization.

The general demographic data and clinical characteristics of the 271 remaining eligible patients are shown in Table 1. Among them, 180 (66.4%) were men, 91 (33.6%) were female, the mean of age was 45.1 ± 13.9 years. The median TBSA burned area was 75.0% (64.3%, 90.0%) and the mean of rBaux score was 147.6 ± 20.6. 107 patients who did not receive an RBC transfusion for > 2 days despite a hemoglobin level < 7 g/dl were assigned to the ultra-restrictive transfusion group; the remaining 164 patients were assigned to the restrictive transfusion group. Compared with the restrictive transfusion group, the proportion of males, the admission MODS scores, and the rate of pulmonary edema were all higher in the ultra-restrictive transfusion group (*p* < 0.05).

**Table 1:** Baseline characteristics, hemoglobin levels and transfusions of ultra-restrictive transfusion group and restrictive transfusion group.

	Total (N = 271)	Ultra-restrictive transfusion (N = 107)	Restrictive transfusion (N = 164)	*p-*value
Baseline characteristics				
Gender (male)	180 (66.4)	80 (74.8)	100 (61.0)	0.019
Age (years)	45.1 ± 13.9	45.7 ± 12.1	44.7 ± 15.0	0.892
≥ 60 years old	38 (14.0)	13 (12.1)	25 (15.2)	0.473
BMI (kg/m^2^)	23.9 (22.2, 25.6)	24.2 (22.0, 26.1)	23.7 (22.4, 25.3)	0.077
TBSA burned%	75.0 (64.3, 90.0)	80.0 (65.0, 91.0)	73.0 (60.0, 85.0)	0.063
Full thickness burned area%	41.2 ± 26.0	41.0 ± 28.3	41.3 ± 24.5	0.771
Type of burn injury				0.259
Flame burn	231 (85.2)	89 (83.2)	142 (86.6)	
Scald burn	14 (5.2)	5 (4.7)	9 (5.5)	
Electrical burn	5 (1.8)	1 (0.9)	4 (2.4)	
Other	21 (7.7)	12 (11.2)	9 (5.5)	
Inhalation injury	199 (73.4)	73 (68.2)	126 (76.8)	0.117
Pulmonary edema	110 (40.6)	55 (51.4)	55 (33.5)	0.003
Admission MODS score	4.0 (2.0, 6.0)	4.0 (3.0, 6.0)	4.0 (2.0, 5.8)	0.038
Admission APACHEII score	13.0 (10.0, 16.2)	13.0 (10.0, 16.2)	13.0 (10.0, 17.0)	0.858
rBaux score	132.7 ± 21.2	134.8 ± 21.3	132.6 ± 21.5	0.402
Underlying disease	62 (22.9)	25 (23.4)	37 (22.6)	0.878
Admission to first operation	4.0 (2.0, 5.0)	4.0 (2.0, 5.0)	4.0 (3.0, 5.0)	0.189
Operation times	4.0 (2.0, 6.0)	4.0 (2.0, 6.0)	3.0 (2.0, 5.0)	0.413
Mechanical ventilation	191 (70.5)	70 (65.4)	121 (73.8)	0.140
Admission hemoglobin(g/dl)	16.9 (15.3, 18.5)	17.0 (15.2, 18.5)	16.7 (15.3, 18.5)	0.817
Hemoglobin levels and transfusions				
Nadir hemoglobin(g/dl)	6.4 (5.7, 7.2)	5.8 (5.3, 6.2)	7.1 (6.3, 7.5)	< 0.001
Mean of the lowest three hemoglobin measurements	7.1 (6.1, 7.9)	6.1 (5.6, 6.7)	7.7 (7.0, 8.1)	< 0.001
Days of hemoglobin < 7 g/dl	1.0 (0.0, 5.0)	6.0 (2.0, 12.0)	0.0 (0.0, 1.0)	< 0.001
Nadir preoperative hemoglobin (g/dl)	8.3 (7.3, 9.4)	6.9 (6.5, 7.6)	8.7 (8.0, 9.6)	< 0.001
Mean preoperative hemoglobin (g/dl)	9.7 (8.8, 10.7)	8.7 (7.7, 9.7)	10.0 (9.3, 11.3)	< 0.001
Nadir postoperative hemoglobin (g/dl)	7.5 (6.8, 8.3)	7.1 (6.3, 7.9)	7.8 (7.0, 8.4)	< 0.001
Mean postoperative hemoglobin (g/dl)	8.8 (8.1, 9.5)	8.2 (7.7, 8.8)	9.1 (8.4, 9.8)	< 0.001
Mean hemoglobin difference between pre- and postoperative (g/dl)	0.8 (0.1, 1.8)	0.6 (− 0.1, 1.3)	0.9 (0.1, 1.8)	0.105
Nadir hemoglobin before RBC transfusion(g/dl)	6.6 (5.8, 7.6)	5.9 (5.3, 6.3)	7.3 (6.5, 7.7)	< 0.001
Mean hemoglobin before RBC transfusion (g/dl)	7.8 (6.8, 8.8)	6.8 (6.2, 7.4)	8.3 (7.8, 9.1)	< 0.001
Total RBC transfusion volume (unit)	15.0 (7.0, 29.5)	11.5 (5.5, 21.5)	17.3 (9.0, 32.5)	0.004
Operating room RBC transfusion volume (unit)	5.0 (0.0, 10.0)	3.5 (0.0, 8.0)	6.0 (2.0, 11.8)	< 0.001
Non-operating room RBC transfusion volume (unit)	9.0 (4.0, 18.0)	7.5 (4.0, 15.0)	10.5 (5.0, 22.0)	0.017
Total plasma transfusion volume (ml)	12,330.0 (5990.0, 21,550.0)	10,410.0 (4330.0, 25,340.0)	12,520.0 (7192.5, 19,755.0)	0.124
Total platelet transfusion volume (unit)	0.0 (0.0, 0.0)	0.0 (0.0, 0.0)	0.0 (0.0, 10.0)	0.069

Data are shown as the median (25th percentile, 75th percentile), mean ± standard deviation or the number of patients (%), as appropriate. *BMI* body mass index, *TBSA* total body surface area, *MODS* multiple organ dysfunction score, *APACHE II* Acute physiology and chronic health evaluation II, *rBaux* revised Baux, *RBC* red blood cell.

### Hemoglobin levels and transfusions of ultra-restrictive transfusion group and restrictive transfusion group

The nadir hemoglobin level was significantly lower in the ultra-restrictive group than in the restrictive transfusion group (5.8 [5.3, 6.2] vs 7.1 [6.3, 7.5] g/dl, *p* < 0.001). Patients in the ultra-restrictive transfusion group had a longer period in which their hemoglobin was < 7 g/dl, while patients in the restrictive transfusion group hardly had such time (6.0 [2.0, 12.0] days vs 0.0 [0.0, 1.0] days). Notably, only 8 patients underwent the ‘ultra-restrictive transfusion’ in the first 2 days postoperative. The nadir preoperative hemoglobin was 6.9 (6.5, 7.6) g/dl in the ultra-restrictive transfusion group and 8.7 (8.0, 9.6) g/dl in the restrictive transfusion group, while the nadir postoperative hemoglobin was 7.1 (6.3, 7.9) g/dl in the ultra-restrictive transfusion group and 7.8 (7.0, 8.4) g/dl in the restrictive transfusion group. The mean hemoglobin difference between pre-and postoperative was 0.6 (− 0.1, 1.3) g/dl in the ultra-restrictive transfusion group and 0.9 (0.1, 1.8) g/dl in the restrictive transfusion group with no significant differences between the two groups (*p* = 0.105).

**Figure 2:**
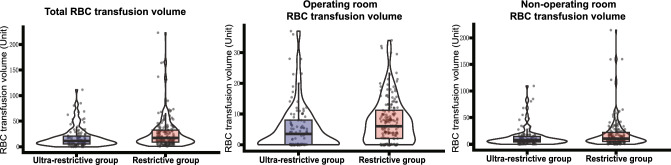
Violin plots of RBC transfusion volumes in two groups. The distributions of RBC transfusion volumes are represented by violin plots. The thicker line in the middle of the box is the median, the upper and lower boundaries of the box represent the third and the first quartile, upper and lower whiskers represent the third quartile plus 1.5 interquartile range (IQR) and first quartile minus 1.5 IQR, and each gray dot represents the RBC transfusion volume of a single patient. The total RBC transfusion volume, operating room RBC transfusion volume and non-operating room RBC transfusion volume in ultra-restrictive transfusion group were significantly lower than restrictive transfusion group. *RBC* red blood cell.

Patients in the ultra-restrictive transfusion group received an RBC transfusion at lower hemoglobin levels (nadir hemoglobin level before RBC transfusion: 5.9 [5.3, 6.3] vs 7.3 [6.5, 7.7] g/dl, *p* < 0.001; mean hemoglobin level before RBC transfusion: 6.8 [6.2, 7.4] vs 8.3 [7.8, 9.1] g/dl, *p* < 0.001). The RBC transfusion volume was significantly lower in the ultra-restrictive group than in the restrictive transfusion group, both inside and outside the operating room (total RBC transfusion volume: 11.5 [5.5, 21.5] vs 17.3 [9.0, 32.5] units, p = 0.004; operating room RBC transfusion volume: 3.5 [0.0, 8.0] vs 6.0 [2.0, 11.8] units, p < 0.001; non-operating room RBC transfusion volume: 7.5 [4.0, 15.0] vs 10.5 [5.0, 22.0] units, p = 0.017). The density distributions of RBC transfusion volumes in two groups are shown in Fig. 2. Furthermore, there were no significant differences in the transfusion volumes of plasma and platelets between the two groups (Table 1).

### Effect of ultra-restrictive transfusion on prognosis

**Figure 3:**
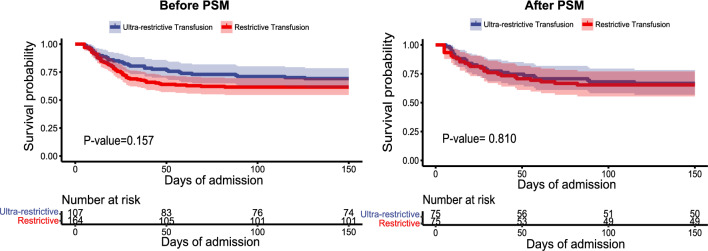
Kaplan–Meier survival curves for patients in ultra-restrictive transfusion group and restrictive transfusion group before and after PSM. The dark blue lines represent the Kaplan–Meier survival curves in ultra-restrictive transfusion group, and the red lines represent the Kaplan–Meier survival curves in restrictive transfusion group. The light blue and light red areas represented the 95% confidence intervals. Before PSM Log-rank *p* = 0.157; After PSM. Log-rank *p* = 0.810. *PSM* propensity score matching.

After the 1:1 PSM, the SD of baseline characteristics were all < 0.2, indicating that the two groups (75 patients per group) were generally balanced (Table S1). The crude hospital mortality had no obvious differences (ultra-restrictive transfusion group 30.8% vs restrictive transfusion group 38.4%, *p* = 0.203). After PSM, the hospital mortality result (33.3% vs 34.7%, *p* = 0.522) was similar to the pre-matching. According to the Kaplan–Meier curves, there were still no significant differences between the two groups (before PSM: *p* = 0.157; after PSM: *p* = 0.810) (Fig. 3). There were also no significant differences in BSI and sepsis incidents between the two groups (BSI before PSM: 68.2% vs 57.9%, *p* = 0.088; BSI after PSM: 65.3% vs 54.7%, *p* = 0.182; sepsis before PSM: 30.8% vs 34.8%, *p* = 0.504; sepsis after PSM: 32.0% vs 26.7%, *p* = 0.473). The crude risks of wound infection and catheter-related infection were significantly higher in the ultra-restrictive transfusion group (*p* < 0.05), but not after PSM (wound infection: 77.3% vs 76.0%, *p* = 0.847; catheter-related infection: 62.7% vs 53.3%, *p* = 0.247). Among surviving patients of both groups, the hospital LOS and WHT had no obvious differences (hospital LOS before PSM: 71.0 [56.0, 91.0] vs 68.5[50.8, 86.0], *p* = 0.683; hospital LOS after PSM: 64.0 [53.3, 84.5] vs 67.0 [51.0, 87.8], *p* = 0.806; WHT before PSM: 55.0 [42.0, 77.0] vs 52.0 [41.8, 66.0], *p* = 0.444; WHT after PSM: 53.5 [39.8, 70.8] vs 49.5 [40.5, 68.0], *p* = 0.962) (Table 2).

**Table 2:** Outcome measures of ultra-restrictive transfusion group and restrictive transfusion.

	Before PSM			After PSM		
	Ultra-restrictive transfusion^a^ (N = 107)	Restrictive transfusion^a^ (N = 164)	*p*-value	Ultra-restrictive transfusion^a^ (N = 75)	Restrictive transfusion^a^ (N = 75)	*p*-value
Primary outcomes						
Hospital mortality	33 (30.8)	63 (38.4)	0.203	25 (33.3)	26 (34.7)	0.863
Secondary outcomes						
BSI	73 (68.2)	95 (57.9)	0.088	49 (65.3)	41 (54.7)	0.182
Sepsis	33 (30.8)	58 (34.8)	0.504	24 (32.0)	20 (26.7)	0.473
Wound infection	90 (84.1)	108 (65.9)	< 0.001	58 (77.3)	57 (76.0)	0.847
Catheter-related infection	74 (69.2)	71 (43.3)	< 0.001	47 (62.7)	40 (53.3)	0.247
Hospital LOS	71.0 (56.0, 91.0)	68.5 (50.8, 86.0)	0.683	64.0 (53.3, 84.5)	67.0 (51.0, 87.8)	0.806
WHT	55.0 (42.0, 77.0)	52.0 (41.8, 66.0)	0.444	53.5 (39.8, 70.8)	49.5 (40.5, 68.0)	0.962

Data are shown as the median (25th percentile, 75th percentile) or the number of patients (%), as appropriate. *PSM* propensity score matching, *BSI* blood stream infection, *LOS* length of stay, *WHT* wound healing time.

Results of the modified Poisson regressions and linear regressions without and with adjustment are shown in Table S2. After adjustment for covariates including gender, BMI, TBSA burned, pulmonary edema, admission MODS score, and the hospital, there were no significant associations between ultra-restrictive transfusion and all prognostic outcome indicators (*p* > 0.05).

### Effect of RBC transfusion threshold < 6 g/dl on prognosis

**Figure 4:**
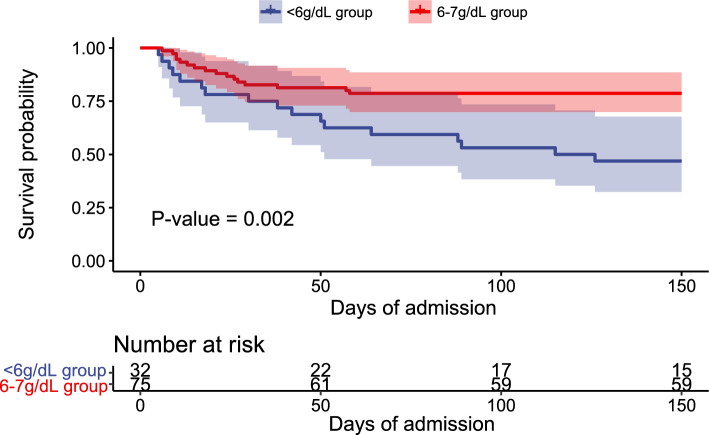
Kaplan–Meier survival curves for patients in 6–7 g/dl group and < 6 g/dl group. The dark blue line represents the Kaplan–Meier survival curve in < 6 g/dl group, and the red line represents the Kaplan–Meier survival curve in 6–7 g/dl group. The light blue and light red areas represented the 95% confidence intervals. Log-rank *p* = 0.02.

Among the ultra-restrictive transfusion group, there were 32 patients did not receive an RBC transfusion for > 2 days despite a hemoglobin level < 6 g/dl. The Baseline characteristics between < 6 g/dl group and 6–7 g/dl group had no significant differences (*p* > 0.05). (Table S3) < 6 g/dl group had a significantly higher hospital mortality than 6–7 g/dl group (53.1% vs 21.3%, *p* = 0.001). The Kaplan–Meier curves also showed significant survival differences between the two groups (*p* = 0.002). (Fig. 4) There were no significant differences in other prognostic outcome indicators between < 6 g/dl group and 6–7 g/dl group (*p* > 0.05) (Table 3).

**Table 3:** Outcome measures of 6–7 g/dl group and < 6 g/dl group.

	6–7 g/dl group (N = 75)	< 6 g/dl group (N = 32)	*p-*value
Primary outcomes			
Hospital mortality	16 (21.3)	17 (53.1)	0.001
Secondary outcomes			
BSI	52 (69.3)	21 (65.6)	0.812
Sepsis	21 (26.7)	13 (40.6)	0.512
Wound infection	66 (88.0)	24 (75.0)	0.092
Catheter-related infection	55 (73.3)	19 (59.4)	0.174
Hospital LOS	67.0 (57.5, 91.0)	83.0 (50.0, 128.5)	0.813
WHT	53.5 (41.3, 74.8)	61.0 (43.0, 89.0)	0.374

Data are shown as the median (25th percentile, 75th percentile) or the number of patients (%), as appropriate. *BSI* blood stream infection, *LOS* length of stay, *WHT* wound healing time.

## Discussion

This retrospective multicenter study investigated the prognostic impacts of ultra-restrictive RBC transfusion in extensive burn patients (TBSA > 50%). According to the comparison between the ultra-restrictive and restrictive RBC transfusion group, we found that an RBC transfusion threshold < 7 g/dl had no significant adverse effects for extensive burn patients while an RBC transfusion threshold < 6 g/dl may increase the mortality risk. Although the potential risk of setting 6–7 g/dl as the transfusion threshold cannot be ruled out, the results will provide a valuable reference for the rational allocation of limited RBC products in extensive burn patients.

TBSA is one of the most important indicators of the severity of burns. Patients with extensive burns often exhibit strong systemic reactions, severe anemia, and high mortality rates after injury. Their treatment often involves massive transfusions of blood products. A large Chinese burn center reported that from 2003 to 2009, the mortality of patients with burns to ≤ 50% of the TBSA was only 0.39%, but 32.19% among those with burns to > 50% of the TBSA^25^. Based on the severity criteria for burns in our country, patients with TBSA more than 50% are specifically classified as critically severe and are usually treated in the burn intensive care unit after the injury^25,26^. Given that this study used mortality and other prognostic outcomes as measures, to prevent less severe patients from masking the impact of transfusion strategies on the prognosis of critically burned patients, this study only included patients with TBSA ≥ 50%. Compared with previous studies of patients with TBSA ≥ 20%, the extensive burn patients in our study had obviously higher mortality and infection rates, longer in-hospital LOSs and WHTs^7,12^.

The RBC transfusion protocol should be designed to ensure optimal clinical outcomes while also minimizing unnecessary RBC transfusions, which may increase the risk of adverse transfusion reactions, as well as waste blood resources and increase costs^6,14^. According to the clinical guidelines from the American Association of Blood Banks in 2016, a total of 31 RCT studies (involving 12,587 participants) compared restrictive transfusion thresholds with liberal transfusion thresholds. Their cross-trial analysis indicated that restrictive RBC transfusion thresholds were not associated with higher rates of adverse clinical outcomes^27^. Subsequently, the TRIBE trial included 347 patients with > 20% TBSA burns to compare these two transfusion strategies (restrictive transfusion threshold set as 7 g/dl; liberal transfusion threshold set as 10 g/dl) in burn patients. The results showed that there were no statistically significant differences in all of the outcome measures, while the restrictive transfusion group received significantly fewer RBC transfusions (3411 vs. 5636 total, median 8 vs 16 units/patient). Based on their estimations, the use of a restrictive blood transfusion strategy in critically ill burn patients in the United States could potentially lead to annual cost savings of approximately $31,543,220 to $47,314,680^7^. Another RCT study included 80 patients with TBSA > 20% and set the restrictive transfusion threshold as 8 g/dl, and the liberal transfusion threshold as 10 g/dl. The mean number of RBC unit transfusions per patient in the restrictive group was significantly lower than in the liberal group (3.28 units vs 5.9 units). They also found no significant difference in mortality rate or other outcome measures between groups^12^. However, in recent years, the shortage of blood resources has led to clinical transfusion thresholds being set lower than those established in the aforementioned studies. As a result, such that some of our patients did not receive RBC transfusions despite a hemoglobin level < 7 g/dl^28^. Especially during the years 2019–2022 with the COVID-19 pandemic, decreased blood donations and increased demand for blood products led to an increase in the number of these patients^28^.

In previous studies, the hemoglobin threshold established in a ‘restrictive transfusion strategy’ was 7.0–8.0 g/dl; therefore, the present study regarded an RBC transfusion threshold < 7 g/dl as ‘ultra-restrictive transfusion’^7,13,14^. Considering the delay between hemoglobin measurement and RBC transfusion, this group consisted of patients who did not receive an RBC transfusion for > 2 days after a hemoglobin level < 7 g/dl was recorded. As expected, patients in the ultra-restrictive transfusion group had significantly lower RBC transfusion thresholds and received significantly smaller RBC volumes. However, the prognostic outcomes did not significantly differ between the two groups with respect to in-hospital mortality, survival time, hospital LOS, and WHT. Infection is another important factor to consider when evaluating RBC transfusion strategies. It is generally believed that transfusion increases the risk of infection through exposure to potentially contaminated blood products and immunomodulatory effects^11,29^. Nevertheless, in the majority of previous RCT studies comparing restrictive transfusion with liberal transfusion, there was no correlation between infection and RBC transfusion strategy in patients^7,30^. Notably, the crude incidences of BSI, wound infection, and catheter-related infection were higher in the ultra-restrictive group. Considering that the infection rates were associated with the culture frequency, which varies widely among different hospitals, we included the hospital in the adjustment together with other baseline characteristics. After PSM or adjusted modified Poisson regressions, there were no significant differences in all types of infection rates between the two groups which was similar to the previous studies. Additionally, among the 271 patients, there were no transfusion-induced adverse events, although the potential risk of transfusion could not be definitively ruled out.

Although our results showed no significant adverse effects of ultra-restrictive RBC transfusion, there is no doubt that the RBC transfusion threshold cannot be lowered indefinitely. When the severity of anemia surpasses the patient's tolerance, it can result in severe consequences such as tissue hypoxia due to insufficient oxygen transport delivery of blood^6,13^. The hypermetabolism, cardiac dysfunction, and severe infections experienced by extensive burn patients may result in a lower tolerance to anemia compared to normal individuals^31^. Thus, we subsequently investigated the prognostic impact of RBC transfusion threshold < 6 g/dl in the ultra-restrictive transfusion group. The results revealed a significant increase in mortality among patients who experienced the absence of RBC transfusion with a hemoglobin level < 6 g/dl. This suggests that lowering the transfusion threshold below 6 g/dl may not be advisable.

Overall, our findings suggest that implementing a transfusion threshold of 6–7 g/dl may alleviate the strain on RBC resources and reduce associated costs in patients with extensive burns, without impacting prognostic outcomes, in comparison to a transfusion threshold of 7–8 g/dl. Although COVID-19 was successfully curtailed, blood scarcity is still a global and long-established problem that involves multiple factors including donor recruitment, blood collection, blood testing, blood processing, blood distribution, blood transfusion management, and so on^17,32^. A modeling study showed that in 2017, the global unmet demand for blood products was equivalent to 1849 units per 100,000 population^33^. The treatment of extensive burn patients requires massive RBC volumes. In our study, although most of the patients had adopted RBC transfusion with a low hemoglobin threshold, the mean RBC transfusion volume was 16 units, much was much higher than the requirement of intensive care unit admission patients and burn patients with TBSA > 20% (ranged from 2 to 4 units)^2,34,35^. When the number of extensive burn patients is relatively large, especially after mass casualty burn incidents (e.g., fire and war), the demand for RBC for the treatment of extensive burns will inevitably put pressure on the allocation of blood resources^36^. At this time, based on our research findings, adopting an RBC transfusion threshold of 6–7 g/dl may help optimize the allocation of blood resources and improve the treatment of more patients. In addition to extensive burns, ultra-restrictive RBC transfusions were also forced to be used in various types of patients. (e.g., patients with tumors, pregnancy-related complications, trauma, gastrointestinal bleeding, sepsis)^17,28^. Considering most clinical studies comparing restrictive and liberal transfusion strategies have reached similar conclusions in different populations, it is believed that our research findings will provide valuable references for subsequent studies on lowering the RBC transfusion threshold in other populations^6,9^.

It is worth noting that the appropriateness of ultra-restrictive RBC transfusion in the perioperative period warrants consideration. Intraoperative blood loss is a major factor contributing to anemia in patients with extensive burns. According to our previous analysis, the median number of surgeries for patients with extensive burns in the three hospitals was 3, with a median intraoperative blood loss of 2541 ml per procedure. Intraoperative blood loss was identified as an independent factor contributing to an increase in RBC transfusion volume for these patients^37^. In the present study, patients demonstrated significantly higher levels of hemoglobin preoperatively and postoperatively compared to their nadir hemoglobin levels. The postoperative hemoglobin levels were basically controlled above 7 g/dl, with only 8 patients subjected to ultra-restrictive RBC transfusion within 2 days after surgery. These results suggested that the perioperative RBC transfusions were not significantly impacted by blood resource constraints, which may be related to the strict preoperative evaluation of patients and the fact that intraoperative RBC transfusion is not limited by hemoglobin threshold. In the previous RCT studies about transfusion strategies in burn patients, RBC transfusions during surgical procedures were similarly not restricted by the defined hemoglobin threshold due to safety considerations^7,12^. Due to the rapid blood loss during surgery leading to a rapid decrease in blood volume, and the potential decrease in patient tolerance to anemia due to surgical stress, restrictions on transfusion thresholds must be used cautiously during intraoperative and perioperative periods. Furthermore, elderly individuals may have decreased physiological function and diminished organ reserves, making them less capable of tolerating low hemoglobin levels compared to young adults^38^. It is important to note that the proportion of elderly patients in our study was relatively small, and therefore, the generalizability of the results to elderly patients also warrants consideration.

This study has some limitations. First, due to the retrospective nature of the study, our grouping method could only be based on whether patients received RBC transfusions at a certain hemoglobin level, rather than their target RBC transfusion thresholds. This grouping method may have reproduced the selection bias of clinicians, who often prioritize the allocation of blood resources to patients in a worse state of illness^39^. Despite being adjusted for covariates related to the different admission characteristics and treatment processes of the two groups, differences in their dynamic conditions could not be completely eliminated. Additionally, the frequency of hemoglobin measurements may impact the grouping, as patients in the ultra-restrictive transfusion group may be misclassified due to inadequate hemoglobin measurement frequency. Second, due to the small number of patients with a transfusion threshold < 6 g/dl, more in-depth statistical analyses (e.g., the adjustment of baseline characteristics and the analysis of mortality risk factor) could not be performed. The specific impact of transfusion threshold < 6 g/dl or lower on extensive burn patients cannot be identified. Third, again due to the limited sample size, the subgroup analysis was not carried out in the present study, thus failing to consider the individualization of transfusion thresholds^31^. Previous studies showed greater anemia tolerance by women than men and by younger adults than older adults^38,40^. Whether ultra-restrictive transfusion has adverse effects in these populations remains uncertain. Fourth, the transfusion strategies in this study were based solely on the hemoglobin level. Other factors including the degree of blood concentration, the causes and progression rate of anemia, as well as the drug usage including iron supplements and erythropoietin should also be considered when choosing the transfusion strategy^31,41^. Fifth, patients were not followed up after discharge from the hospital; thus, the long-term complications of ultra-restrictive transfusion still cannot be ruled out^42,43^. Future large-sample and multicenter studies are warranted to address these limitations and determine the optimal RBC transfusion protocol. Patients should be classified according to various clinical characteristics that may affect anemia tolerance (e.g., gender, age, TBSA), and the lowest hemoglobin threshold that each subgroup of patients can tolerate without affecting prognosis should be determined. In addition to focusing on outcome indicators during hospitalization, long-term hemoglobin levels and cardiopulmonary function after discharge should also be taken into consideration.

## Conclusions

Our results showed that, for extensive burns, ultra-restrictive transfusion with an RBC transfusion threshold < 7 g/dl had no significant effect on hospital mortality, hospital LOS, WHT, and risk of infection. However, an RBC transfusion threshold < 6 g/dl may increase the mortality risk. When the blood supply is tight, it is acceptable to adopt an RBC transfusion threshold of < 7 g/dl but not < 6 g/dl. In addition, prospective cohort multicenter studies to better define the use of restrictive and ultra-restrictive RBC transfusion strategies are recommended.

### Supplementary Information

Supplementary Tables.

## Data Availability

The datasets used in this study are not publicly available due to they contain the personal health information and important privacy of each patient, but are available from the corresponding authors on reasonable request.

## References

[1] Liu NT (2020). Quantifying the effects of wound healing risk and potential on clinical measurements and outcomes of severely burned patients: A data-driven approach. Burns.

[2] Wu G (2016). Blood transfusions in severe burn patients: Epidemiology and predictive factors. Burns.

[3] Loftus TJ (2018). The postinjury inflammatory state and the bone marrow response to anemia. Am. J. Respir. Crit. Care Med..

[4] McEvoy MT, Shander A (2013). Anemia, bleeding, and blood transfusion in the intensive care unit: Causes, risks, costs, and new strategies. Am. J. Crit. Care.

[5] Palmieri TL, Sen S, Falwell K, Greenhalgh DG (2011). Blood product transfusion: Does location make a difference?. J. Burn Care Res..

[6] Carson JL (2016). Transfusion thresholds and other strategies for guiding allogeneic red blood cell transfusion. Cochrane Database Syst. Rev..

[7] Palmieri TL (2017). Transfusion requirement in burn care evaluation (TRIBE): A multicenter randomized prospective trial of blood transfusion in major burn injury. Ann. Surg..

[8] Amatto M, Acharya H (2016). Secondary hemochromatosis as a result of acute transfusion-induced iron overload in a burn patient. Burns Trauma.

[9] Carson JL (2011). Liberal or restrictive transfusion in high-risk patients after hip surgery. N. Engl. J. Med..

[10] Kwan P, Gomez M, Cartotto R (2006). Safe and successful restriction of transfusion in burn patients. J. Burn Care Res..

[11] Palmieri TL (2006). Effect of blood transfusion on outcome after major burn injury: A multicenter study. Crit. Care Med..

[12] Salehi SH, Daniali M, Motaghi P, Momeni M (2021). The best strategy for red blood cell transfusion in severe burn patients, restrictive or liberal: A randomized controlled trial. Burns.

[13] Carson JL (2021). Transfusion thresholds for guiding red blood cell transfusion. Cochrane Database Syst. Rev..

[14] Mueller MM (2019). Patient blood management: Recommendations from the 2018 Frankfurt consensus conference. JAMA.

[15] Chen L (2022). Blood transfusion risk prediction in spinal tuberculosis surgery: Development and assessment of a novel predictive nomogram. BMC Musculoskelet. Disord..

[16] Jian J (2022). Determining transfusion use in major burn patients: A retrospective review and analysis from 2009 to 2019. Burns.

[17] Faria I, Thivalapill N, Makin J, Puyana JC, Raykar N (2022). Bleeding, hemorrhagic shock, and the global blood supply. Crit. Care Clin..

[18] Luo J (2022). The effect and evaluation of the third military medical university fluid resuscitation formula. Evid. Based Complement. Altern. Med..

[19] Marshall JC (1995). Multiple organ dysfunction score: A reliable descriptor of a complex clinical outcome. Crit. Care Med..

[20] Knaus WA, Draper EA, Wagner DP, Zimmerman JE (1985). APACHE II: A severity of disease classification system. Crit. Care Med..

[21] Prasad A, Thode HC, Singer AJ (2020). Predictive value of quick SOFA and revised Baux scores in burn patients. Burns.

[22] Hu Y (2021). Epidemiology and outcomes of bloodstream infections in severe burn patients: A six-year retrospective study. Antimicrob. Resist. Infect. Control.

[23] Singer M (2016). The third international consensus definitions for sepsis and septic shock (sepsis-3). JAMA.

[24] Fink DS (2017). Deployment and alcohol use in a military cohort: Use of combined methods to account for exposure-related covariates and heterogeneous response to exposure. Am. J. Epidemiol..

[25] Chen J (2013). Characteristics of burn deaths from 2003 to 2009 in a burn center: A retrospective study. Burns Trauma.

[26] Zheng XF (2020). Management of combined massive burn and blast injury: A 20-year experience. Burns.

[27] Carson JL (2016). Clinical practice guidelines from the AABB: Red blood cell transfusion thresholds and Storage. JAMA.

[28] Kheirbek T, Martin TJ, Wakeley ME, Lueckel SN, Adams CA (2022). Safety and feasibility of ultra-restrictive transfusion protocol as a blood-preservation strategy during shortage crises. Rhode Island Med. J..

[29] Jeschke MG (2007). Blood transfusions are associated with increased risk for development of sepsis in severely burned pediatric patients. Crit. Care Med..

[30] Palmieri TL (2021). Transfusion and infections in the burn patient. Surg. Infect..

[31] Palmieri TL (2019). Burn injury and blood transfusion. Curr. Opin. Anaesthesiol..

[32] Barro L (2018). Blood transfusion in sub-Saharan Africa: Understanding the missing gap and responding to present and future challenges. Vox Sanguinis.

[33] Roberts N, James S, Delaney M, Fitzmaurice C (2019). The global need and availability of blood products: A modelling study. Lancet. Haematol..

[34] Blet A (2022). Association between in-ICU red blood cells transfusion and 1-year mortality in ICU survivors. Crit. Care.

[35] Hébert PC (1999). A multicenter, randomized, controlled clinical trial of transfusion requirements in critical care. Transfusion requirements in critical care investigators, canadian critical care trials group. N. Engl. J. Med..

[36] Tian H (2018). Epidemiologic and clinical characteristics of severe burn patients: Results of a retrospective multicenter study in China, 2011–2015. Burns Trauma.

[37] Dq D (2023). Multicenter retrospection and analysis of influencing factors on blood transfusion in patients with extensive burns. Chin. J. Burns Wounds.

[38] Simon GI (2019). Impacts of aging on anemia tolerance, transfusion thresholds, and patient blood management. Transfus Med. Rev..

[39] Nunan D, Heneghan C, Spencer EA (2018). Catalogue of bias: Allocation bias. BMJ Evid. Based Med..

[40] Visagie M (2019). Greater anemia tolerance among hospitalized females compared to males. Transfusion.

[41] Kranenburg FJ, Arbous MS, Le Cessie S, Van der Bom JG (2015). The, “grey area” of the transfusion practice in the intensive care unit. Intens. Care Med. Exp..

[42] Palmieri TL (2019). Restrictive transfusion strategy is more effective in massive burns: Results of the TRIBE multicenter prospective randomized trial. Mil. Med..

[43] Roubinian NH (2019). Long-term outcomes among patients discharged from the hospital with moderate anemia: A retrospective cohort study. Ann. Intern. Med..

