# Supplementary material for: Ultra-restrictive red blood cell transfusion strategies in extensively burned patients
Source: Sci Rep. 2024 Feb 3;14:2848. doi: 10.1038/s41598-024-52305-y (PMC10838330; doi:10.1038/s41598-024-52305-y)
Supplement: Supplementary file 1 — Supplementary Tables. [file 41598_2024_52305_MOESM1_ESM.docx]

**Ultra-Restrictive Red Blood Cell Transfusion Strategies in Extensively Burned Patients**

**Yiran Wang^a, b †^, Zhikang Zhu^a, b †^, Deqing Duan^c^, Wanting Xu^d^, Zexin Chen^e^, Tao Shen^a^, Xingang Wang^a, b, *^, Qinglian Xu^d,^** ***, Hongyan Zhang^c,^** ***, Chunmao Han^a, b, *^**

**Table S1** Baseline characteristics of ultra-restrictive transfusion group and restrictive transfusion group after PSM

| **Baseline characteristics** | **Ultra-restrictive Transfusion**  **(N=75)** | **Restrictive Transfusion**  **(N=75)** | ***p-*value** | **SMD** |
| --- | --- | --- | --- | --- |
| Gender(male) | 55 (73.3) | 52 (69.3) | 0.588 | -0.092 |
| Age(years) | 46.0 (36.0, 51.0) | 46.0 (35.0, 53.5) | 0.875 | -0.051 |
| BMI(kg/㎡) | 23.8 (22.5, 25.4) | 24.0 (22.0, 25.7) | 0.666 | 0.083 |
| TBSA burned% | 78.0 (65.0, 90.0) | 80.0 (65.5, 91.0) | 0.592 | 0.062 |
| Full thickness burned area% | 41.5(17.0, 60.0) | 33.0(15.0 55.0) | 0.470 | 0.104 |
| Type of burn injury |  |  | 0.648 |  |
| Flame burn | 66 (88.0) | 64 (85.3) |  | -0.071 |
| Scald burn | 2 (2.7) | 5 (6.7) |  | 0.190 |
| Electrical burn | 2 (2.7) | 1 (1.3) |  | -0.139 |
| Other | 5 (6.7) | 5 (6.7) |  | 0.000 |
| Inhalation injury | 54 (72) | 51 (68) | 0.593 | -0.086 |
| Pulmonary edema | 32 (42.7) | 33 (44) | 0.869 | 0.027 |
| Admission MODS score | 4.0 (2.0, 6.0) | 4.0 (2.0, 6.0) | 0.581 | 0.041 |
| Admission APACHEII score | 13.5±5.9 | 14.0±6.5 | 0.578 | 0.092 |
| rBaux score | 133.4±21.6 | 133.8±22.9 | 0.912 | 0.018 |
| Underlying disease | 14 (18.7) | 13 (17.3) | 0.832 | -0.032 |
| Admission to first operation | 4.0(0.0,5.0) | 4.0 (2.0,5.0) | 0.648 | 0.170 |
| Operation times | 3.0 (1.5, 5.0) | 3.0 (2.0, 5.0) | 0.893 | -0.004 |
| Mechanical ventilation | 27 (36) | 28 (37.3) | 0.865 | -0.028 |
| Admission hemoglobin(g/dL) | 16.5(15.1, 18.5) | 17.1 (15.3, 18.5) | 0.692 | -0.033 |
| Hospital |  |  | 0.917 |  |
| 1 | 48 (64) | 50 (66.7) |  | 0.061 |
| 2 | 11 (14.7) | 11 (14.7) |  | 0.000 |
| 3 | 16 (21.3) | 14 (18.7) |  | -0.073 |

Data are shown as the median (25th percentile, 75th percentile), mean±standard deviation or the number of patients (%), as appropriate. PSM: propensity score matching; SMD: Standardized difference; BMI: body mass index; TBSA: total body surface area; MODS: multiple organ dysfunction score; APACHE II: Acute physiology and chronic health evaluation II; rBaux: revised Baux.

**Table S2** Association between ultra-restrictive transfusion and prognostic outcome indicators

| **Variables** | **Unadjusted analysis** | | **Adjusted analysis^a^** | |
| --- | --- | --- | --- | --- |
| **Primary outcomes** | **RR (95% CI)** | ***p-*value** | **RR (95% CI)** | ***p-*value** |
| Hospital mortality | 0.803(0.569-1.132) | 0.210 | 0.859(0.594-1.242) | 0.418 |
| **Secondary outcomes** | **RR (95% CI)** | ***p-*value** | **RR (95% CI)** | ***p-*value** |
| BSI | 1.178(0.980-1.415) | 0.081 | 1.083(0.901-1.322) | 0.414 |
| Spesis | 0.887(0.624-1.263) | 0.507 | 1.046(0.711-1.539) | 0.819 |
| Wound infection | 1.277(1.113-1.466) | <0.001 | 1.052(0.913-1.212) | 0.483 |
| Catheter-related infection | 1.597(1.287-1.983) | <0.001 | 1.098(0.890-1.353) | 0.383 |
|  | **β (95% CI)** | ***p-*value** | **β (95% CI)** | ***p-*value** |
| Hospital LOS | 2.20(-9.58-13.98) | 0.713 | -7.43(-18.11-9.89) | 0.202 |
| WHT | 4.15(-5.03-13.33) | 0.374 | -3.36(-12.11-5.38) | 0.449 |

RR was determined from modified Poisson regression analysis; β was determined from linear regression analysis. a. RR and β were adjusted for gender, BMI, TBSA burned, pulmonary edema, admission MODS score and hospital. RR: risk ratio; BSI: blood stream infection; LOS: hospital length of stay; WHT: wound healing time; BMI: body mass index; TBSA: total body surface area.

**Table S3** Baseline characteristics of 6-7 g/dL group and ＜6g/dL group

| **Baseline characteristics** | **＜6g/dL group**  **(N=32)** | **6-7 g/dL group**  **(N=75)** | ***p-*value** |
| --- | --- | --- | --- |
| Gender(male) | 26 (81) | 54 (72) | 0.313 |
| Age(years) | 47.8 ± 11.5 | 44.8±12.9 | 0.236 |
| BMI(kg/㎡) | 24.5 ± 2.5 | 24.4 ± 3.3 | 0.946 |
| TBSA burned% | 82.0 (66.0, 92.0) | 80.0 (65.0, 90.0) | 0.483 |
| Full thickness burned area% | 47.5 (24.5, 76.8) | 40.0 (15.0, 59.0) | 0.162 |
| Type of burn injury |  |  | 1.000 |
| Flame burn | 27 (84) | 62 (83) |  |
| Scald burn | 1 (3) | 4 (5) |  |
| Electrical burn | 4 (13) | 8 (11) |  |
| Other | 0 (0) | 1 (1) |  |
| Inhalation injury | 23 (72%) | 50 (67%) | 0.596 |
| Pulmonary edema | 14 (44%) | 41 (55%) | 0.301 |
| Admission MODS score | 5.0 (2.8, 6.0) | 4.00 (3.0, 6.5) | 0.856 |
| Admission APACHEII score | 11.9 (8.7, 16.7) | 13.3 (10.3, 16.0) | 0.617 |
| rBaux score | 139 ± 23 | 133 ± 21 | 0.200 |
| Underlying disease | 11 (34) | 14 (19) | 0.079 |
| Admission to first operation | 3.0 (2.0, 5.0) | 4.0 (2.0, 5.0) | 0.978 |
| Operation times | 3.5 (1.0, 6.5) | 4.0 (2.0, 5.5) | 0.661 |
| Mechanical ventilation | 23 (72%) | 47 (63%) | 0.359 |
| Admission hemoglobin(g/dL) | 16.4 (14.9, 18.4) | 17.2 (15.4, 18.6) | 0.364 |
| Hospital |  |  | 0.387 |
| 1 | 21 (66) | 58 (77) |  |
| 2 | 4 (13) | 7 (9) |  |
| 3 | 7 (22) | 10 (13) |  |

Data are shown as the median (25th percentile, 75th percentile), mean±standard deviation or the number of patients (%), as appropriate. BMI: body mass index; TBSA: total body surface area; MODS: multiple organ dysfunction score; APACHE II: Acute physiology and chronic health evaluation II; rBaux: revised Baux.
